# Periapical Status and Quality of Root canal Fillings and Coronal Restorations in Iranian Population

**Published:** 2010-05-20

**Authors:** Saeed Asgary, Bahareh Shadman, Zohreh Ghalamkarpour, Arash Shahravan, Jamileh Ghoddusi, Ali Bagherpour, Alireza Akbarzadeh Baghban, Maryam Hashemipour, Madjid Ghasemian Pour

**Affiliations:** 1. Department of Endodontics, Iranian Center for Endodontic Research, Dental Research Center, Dental School, Shahid Beheshti University of Medical Sciences, Tehran, Iran.; 2. Researcher, Iran Center for Dental Research, Shahid Beheshti University of Medical Sciences, Tehran, Iran.; 3. Endodontist, Iranian Center for Endodontic Research, Dental School, Shahid Beheshti University of Medical Sciences, Tehran, Iran.; 4. Department of Endodontics, Oral and Dental Diseases Research Center, Dental School, Kerman University of Medical Sciences, Kerman, Iran.; 5. Department of Endodontics, Dental School, Mashad University of Medical Sciences, Mashad, Iran.; 6. Department of Oral Radiology, Dental School, Mashad University of Medical Sciences, Mashad, Iran.; 7. Department of Biostatistics, Paramedical School, Shahid Beheshti University of Medical Sciences, Tehran, Iran.; 8. Department of Oral Medicine, Oral and Dental Diseases Research Center, Dental School, Kerman University of Medical Sciences, Kerman, Iran.; 9. Orthodontist, Iran Center for Dental Research, Shahid Beheshti University of Medical Sciences, Tehran, Iran.

**Keywords:** Apical Periodontitis, Coronal Restoration, Endodontic Outcomes, Epidemiology, Root Fillings

## Abstract

**INTRODUCTION:**

This cross-sectional survey determined the dental prevalence of apical periodontitis (AP) in selected Iranian population, and evaluated the influence of the quality of root canal treatment (RCT) and their coronal restorations (CR) on the periapical status.

**MATERIALS AND METHODS:**

A total of 1064 panoramic radiographies were evaluated by two observers during 2009. The quality of RCT i.e. length/density of root fillings and CR in addition to periapical status of endodontically treated teeth were recorded. Their interrelationship was analyzed by Chi-squared, logistic regression and Spearman's rho statistics. Hosmer and Lemeshow tests were used for assessing fitness of logistic regression model and one sample k-s test was used for evaluating of normality of the data.

**RESULTS:**

Our results showed that 527 teeth (52%) of the endodontically treated teeth presented with AP radiographically. The percentages of teeth which fulfilled the criteria of an acceptable RCT or CR radiographically were 42.3 and 62.5 respectively. Incidence of AP among teeth with acceptable RCT (29.1%) was significantly lower than those suffering from unacceptable RCT (68.8%) (P<0.001). Moreover, adequate CR demonstrated a significantly better periapical status (58.6%) compared to teeth with inadequate CR (30.3%) (P<0.001). The incidence of AP ranged from 25.6% (good qualities) to 79.5% (bad qualities) (P<0.001). Cases with both unacceptable RCT/CR were 11 times more likely to have AP than cases with acceptable RCT/CR. The quality of RCT and CR were found to impact the periapical health of endodontically treated teeth.

**CONCLUSION:**

There are a significant high number of technically unacceptable endodontic and restorative treatments in Iran; therefore considerable efforts are needed to improve the standards of endodontic and restorative treatments.

## INTRODUCTION

Currently, root canal therapy (RCT) either with advanced techniques and materials or with conventional methods is a predictable procedure with a high degree of success [1]; however, failures may occur after treatment. RCT outcome is mainly assessed either by functionality of the tooth involved, presence of signs and symptoms, radiographic changes, and/or histopathological evaluation of the excised tissue [2].

Outcomes studies can be designed using two major approaches: case-controlled or epidemiologic study. The outcome of RCT in case-controlled studies has yielded success rates up to 98% [3]. The high rates of success reported in such studies is from a relatively small number of endodontic treatment cases and controls which are usually carried out by endodontic specialists or undergraduate students under strict operating conditions in a university clinic [4][5][6]. Therefore such studies may not represent the reality of treatment carried out in the general practitioners’ clinic [7].

Epidemiologic surveys assess a large number of RCTs performed by both general practitioners and endodontists; therefore they will yield success rates that more realistically represent the treatment outcomes in the general population [8]. During the last decades, cross-sectional studies of the prevalence of apical periodontitis (AP) have been performed in several countries. Majority of these epidemiologic radiographic studies were conducted in European populations and mainly assessed the prevalence of AP after treatment [9]. Unfortunately, a high percentage of inappropriate RCTs chiefly performed by general practitioners have been reported in many surveys; i.e. 24.5 to 65.8% of the endodontically treated teeth presented with AP [10][11][12][13][14][15][16]. These studies have shown an association between the quality of RCT and AP, and have concluded that an improvement in the quality of RCT in general dental practice is necessary in order to promote periapical dental health [15].

Recently, it has been suggested that quality of the coronal restoration (CR) may have greater bearing on the periapical status than the RCT quality [11]. Therefore, prevention of recontamination by a proper coronal restoration is a major requirement of current endodontic treatment [17]. Temporary restorations and obturated root canals are not impervious to bacteria and their by-products, and lower success rates have been reported when improper CRs were inserted [11].

Endodontic epidemiological studies are important because they a complete picture of the distribution and prevalence of AP and its determinants, including treatment outcome in different populations (evaluated by the presence or absence of AP) [9]. They also help us develop better diagnostic methods, superior treatment, and post-treatment advice [18]. Moreover these data play an important role in case selection and treatment planning. It also enables the clinician to make more predictable evidence-based decisions regarding the long-term prognosis of RCT [2].

The quality of RCT/CR is considered an important prognostic factor for endodontic treatment outcome. There is no available data about the prevalence and technical standard of RCT/CR, and the occurrence of AP in Iran. Therefore the aim of the present study was to record the prevalence of AP and quality of endodontic treatment, coronal restoration and their inter-relationship in individuals seeking examination and treatment in three Iranian dental schools. The treatment outcomes were based on radiographic examination.

## MATERIALS AND METHODS

The OPG radiographs of 1064 patients presenting consecutively as new patients seeking routine dental care in three Iranian dental schools (Shahid Beheshti Dental School, Tehran, Kerman Dental School, Kerman, and Mashad Dental School, Mashad, Iran) during 2008 to 2009 were studied. These geographical sites were chosen because they were considered to reflect the centre, south, and north regions of Iran, respectively. The inclusion criteria in the study were patients with ten or more remaining natural teeth who attended the school for the first time. Patients <18 years old and those who received endodontic treatment during the last 2 years were not included. The study was approved by Iranian Center for Endodontic Research and the Ethics Committee of Shahid Beheshti Medical University, Tehran, Iran.

In each dental school, all OPG radiographs that were taken by one radiologist in the department were chosen (using the OPG machine, Planmeca, Helsinki, Finland).

Teeth were categorized as endodontically treated teeth if they had been obturated with a radiopaque material in the pulp chamber and/or in the root canal(s). The parameters used were as follows: length and density of root filling, overall quality of root filling based on length/density of root filling, adequacy of coronal restoration, and presence/absence and the size of AP. These parameters were evaluated based on those described by other researchers [19][20][21][22] (Table 1).

**Table 1:** Radiographic variables and diagnostic categories

**Parameters**	**Registrations and codes**
***Apical periodontitis*** ***(Orstavik et al. 1986)***	1 = Absence (Normal periapical structures; or small changes in bone structure)
	2 = Presence (Changes in bone structure with some mineral loss; apical periodontitis with well-defined radiolucent area; or extensive/severe periodontitis with exacerbating features
***Size of AP^a^***	1= <3 mm
	2= >3 mm and <5 mm
	3= >5 mm
***Length of root fillingb*** ***(DeMoor et al. 2000)***	1= Adequate (<2 mm from, or flush with, the radiographic apex)
	2= Inadequate (>2 mm from the radiographic apex or overextended)
***Density of root filling^b^*** ***(Dugas et al. 2003)***	1= Adequate (Uniform density and adaptation of the filling to the root canal walls)
	2= Inadequate (Visible canal space laterally along the filling; voids within the filling mass; or identifiable untreated canal)
***Coronal restorations *** ***(Siqueira et al. 2005)***	1 = Adequate (radiographically intact restoration with no signs of leakage)
	2 = Inadequate (radiographic signs of overhangs; open margins/ recurrent decay; presence of temporary coronal restoration; or no coronal restoration)

^a^ If a multirooted tooth presented with different periapical status at different roots, the root canal with the most severe periapical condition was categorized.

^b^ In cases of multirooted teeth, not all root canal fillings of such teeth were assessed separately but only the canal with the worst technicall obturation quality.

Two observers examined all the radiographs independently in 2009. They were examined by scoring thirty OPGs that were not included in the study. Scoring was repeated after one month to evaluate intra-rater reliability. Cohen’s Kappa statistics showed that the radiographic inter-rater reliability was good (κ=0.84). In case of disagreement, consensus was reached by dialogue between operators. Intra-rater reliability yielded a perfect score in terms of presence/absence of AP (κ=0.92).

SPSS software (version 15) was used for statistical analysis. Data were analyzed using the Chi-square test, logistic regression model (Hosmer and Lemeshow Test was used for assessing fitness of logistic regression model) and Spearman's rho correlation coefficient (one sample k-s test was used for evaluating normality of the data). Significance level was established at 5%.

## RESULTS

The average patient age was 31.88±12.95 years (male 34.10±13.93 and female 30.28±11.96). Out of the 1064 individuals, 445 were male (41.8%) and 619 were female (58.2%). Overall, the patients had a total of 28463 functional teeth (mean 26.75). Males had fewer natural remaining teeth than females (25.42±6.33 vs. 27.70±4.97) (P < 0.001). Similarly, the average number of root filled teeth was lower for men (0.89±1.53 vs. 1.00±1.59) (P =0.29).

The number of missing teeth according to age group is presented in Table 2. The number of missing teeth per person significantly increased with age (r= 0.659, P < 0.001).

**Table 2:** Statistical indices of the remaining and missing teeth in studied samples

****	**Age Groups**	**Number**	**Percentiles**							**Mean**	**SD**
			**5**	**10**	**25**	**50**	**75**	**90**	**95**		
**Remaining Teeth**	**≤ 20**	175	26.0	28.0	30.0	31.0	32.0	32.0	32.0	30.31	2.98
	**21-30**	473	22.0	25.0	28.0	30.0	32.0	32.0	32.0	29.19	3.62
	**31-40**	146	17.0	18.7	23.7	27.0	30.0	31.3	32.0	25.95	5.02
	**41-50**	148	13.0	14.0	18.0	22.0	26.0	29.0	29.0	21.57	5.18
	**51-60**	83	10.0	11.0	16.0	20.0	24.0	27.0	28.0	19.73	5.62
	**> 60**	39	10.0	12.0	16.0	18.0	22.0	25.0	28.0	18.82	4.63
**Missing Teeth**	**≤ 20**	175	0.0	0.0	0.0	1.0	2.0	4.0	6.0	1.69	2.98
	**21-30**	473	0.0	0.0	0.0	2.0	4.0	7.0	10.0	2.81	3.62
	**31-40**	146	0.0	0.7	2.0	5.0	8.2	13.3	15.0	6.05	5.02
	**41-50**	148	3.0	3.0	6.0	10.0	14.0	18.0	19.0	10.43	5.18
	**51-60**	83	4.0	5.0	8.0	12.0	16.0	21.0	22.0	12.27	5.62
	**> 60**	39	4.0	7.0	10.0	14.0	16.0	20.0	22.0	13.18	4.63

A total of 441 individuals (41.4% of OPGs) had one or more endodontically treated teeth (1013 teeth, 3.56% of total teeth). More than half of these teeth showed AP (n=527; 52.0%); prevalence of evaluated parameters are shown in Table 3.

**Table 3:** Distribution of studied variables

**Variable**	**Condition**	**Number**	**Percent**
**Length of root filling**	Adequate	535	52.8
	Inadequate	478	47.2
**Density of root filling**	Adequate	543	53.6
	Inadequate	470	46.4
**Quality of RCT**	Acceptable	429	42.3
	Unacceptable	584	57.7
**Apical periodontitis**	Absence	486	48.0
	Presence	527	52.0
**Size of AP**	<3 mm	472	89.6
	>3 mm and <5 mm	46	8.7
	>5 mm	9	1.7
**Coronal restorations (CR)**	Adequate	633	62.5
	Inadequate	380	37.5

The prevalence of endodontically treated teeth and those with AP based on tooth type and sex is presented in Table 4. It was determined that 56% of the endodontically treated teeth were in the maxilla and 44% in the mandible. Mandibular molars had the highest incidence of RCT (25%), followed by maxillary premolars (19%) and the mandibular canines had the lowest incidence of root filled teeth (1%) (P<0.001).

**Table 4:** Distribution of endodontically treated teeth and those with apical periodontitis (AP) by tooth type and sex

**Tooth Type**		**Endodontically Treated Teeth**						**Endodontically Treated Teeth with AP**					
		**Total **		**Male **		**Female **		**Total**		**Male**		**Female**	
		**n **	**%**	**n **	**%**	**n**	**% **	**n**	**%**	**n **	**%**	**n**	**%**
**Maxilla**	Incisors	146	25.6	45	19.7	101	29.6	76	27.7	22	19.5	54	33.5
	Canines	63	11.1	24	10.5	39	11.4	35	12.8	14	12.4	21	13.0
	Premolars	196	34.4	87	38.0	109	32.0	82	29.9	38	33.6	44	27.3
	Molars	165	28.9	73	31.9	92	27.0	81	29.6	39	34.5	42	26.1
	**Subtotal**	**570**	**100**	**229**	**100**	**341**	**100**	**274**	**100**	**113**	**100**	**161**	**100**
**Mandible**	Incisors	17	3.8	7	4.2	10	3.6	12	4.7	4	4.1	8	5.2
	Canines	12	2.7	4	2.4	8	2.9	7	2.8	3	3.1	4	2.6
	Premolars	160	36.1	62	36.9	98	35.6	79	31.2	30	30.6	49	31.6
	Molars	254	57.3	95	56.5	159	57.8	155	61.3	61	62.2	94	60.6
	**Subtotal**	**443**	**100**	**168**	**100**	**275**	**100**	**253**	**100**	**98**	**100**	**155**	**100**

A clear correlation was found between prevalence of AP and length/density of root filling of the endodontically treated teeth (P<0.001) (Table 5). Teeth that had root fillings with adequate length/density (acceptable RCT) were tested against any other combination of unacceptable RCTs (Table 5). Both length and density were found to be adequate in 429 teeth (42.3%); interestingly 29.1% of these teeth had AP, significantly less than any other combination of parameters (P<0.001). In the cases of unacceptable RCT, AP was present in 68.8% of teeth. The multivariate logistic regression analysis which combined two independent variables (length and density) of root fillings for the variable AP, confirmed that adequate length (OR=3.17, 95% CI: 2.33-4.32) and density (OR=2.67, 95% CI: 1.96-3.64) of root canal fillings significantly affected the periapical status (Table 6).

**Table 5:** Distribution of apical periodontitis of endodontically treated teeth in relation to the quality of root canal therapy (RCT), coronal restoration (CR), and their combination.

**Parameter**	**Total**		**Apical periodontitis**		**P value**
	**n**	**%**	**n**	**%**	
Endodontically treated teeth (n=1064)	1013	100	527	52.0	-
Adequate length/Adequate density of root filling (Acceptable RCT)	429	42.3	125	29.1	0.001
Adequate length/Inadequate density of root filling (Unacceptable RCT)	106	10.5	55	51.9	
Inadequate length/Adequate density of root filling (Unacceptable RCT)	114	11.3	64	56.1	
Inadequate length/Inadequate density of root filling (Unacceptable RCT)	364	35.9	283	77.7	
Unacceptable RCT	584	57.7	402	68.8	-
Adequate CR	633	62.5	262	41.4	0.001
Inadequate CR	380	37.5	265	69.7	
Acceptable RCT/Adequate CR	332	32.8	85	25.6	0.001
Acceptable RCT/Inadequate CR	97	9.6	40	41.2	
Unacceptable RCT/Adequate CR	301	29.7	177	58.8	
Unacceptable RCT/Inadequate CR	283	27.9	225	79.5	

**Table 6:** Logistic regression output showing the influence of two independent variables, the length of root filling and the density of root filling on the dependent variable apical periodontitis

	**B **	**SE **	**Wald **	**df **	**Sig. **	**Exp (B) (Odds ratio) **	**95% CI for EXP(B) **	
							**Lower **	**Upper **
**Length of root filling**	1.15	0.15	53.18	1	0.000	3.17	2.32	4.32
**Density of root filling**	0.98	0.15	38.48	1	0.000	2.67	1.96	3.64
**Constant**	-0.89	0.10	79.46	1	0.000	0.40		

The relationship between quality of CR and AP is also presented in Table 5. Apical periodontitis was present in approximately 40% of teeth which were treated properly, compared to 70% which were treated improperly (P<0.001).

The parameters for the combined quality of the CR and RCT are also shown in Table 5. Both these variables were only adequate in 332 teeth (32.8%), and approximately one-forth of these teeth (25.6%) had AP (Table 5). When tested against other combinations of the quality of parameters, the acceptable CR and RCT combined category was significantly better than the others (P<0.001). Conversely, when the CR and RCT were unacceptable (283 teeth), 79.5% of the endodontically treated teeth had AP (P<0.001). Finally, the multivariate logistic regression analysis confirmed that the quality of RCT (OR=4.54, 95% CI: 3.43-6.01) and the quality of CR (OR=2.43, 95% CI: 1.82-3.25) had a significant influence on the AP (Table 7). The odds of AP/normal periodontal status in cases with both unacceptable RCT/CR was >11 times greater compared to cases with acceptable RCT/CR.

**Table 7:** Logistic regression output of the influence of two independent variables: the quality of RCT and the quality of the coronal restoration (CR) on the dependent variable, apical periodontitis

	**B **	**SE**	**Wald **	**df **	**Sig. **	**Exp (B) (Odds ratio) **	** 95% CI for EXP (B) **	
							**Lower **	**Upper **
**Quality of RCT**	1.51	0.14	112.98	1	0.000	4.54	3.43	6.01
**Quality of CR**	0.89	0.14	36.63	1	0.000	2.43	1.82	3.25
**Constant**	-1.11	0.11	93.15	1	0.000	0.32		

## DISCUSSION

Endodontic epidemiological studies and clinical trials are the two major approaches for evaluating treatment outcomes of RCT. However endodontic literature has not often conducted epidemiological surveys [18]. A review of the current literature revealed that only ~1% of the articles published were endodontic epidemiologic surveys [9]. Epidemiological surveys demonstrate what is achieved with endodontic treatment in general practice, whilst studies from controlled environments are usually carried out by specialists and disclose the potential outcome of RCT rather than its realistic outcome in the general population [9]. Consequently, the success rates of RCT carried out in clinical case-controlled studies are significantly higher than those observed in epidemiologic surveys [22].

The main limitations of cross-sectional epidemiological studies are researchers’ inability to randomize and standardize the experiment and to show the dynamic nature of periapical healing [23]; the results must therefore be interpreted with caution. These studies have advantages such as larger study populations, longer follow-up periods and are often protected from bias [18]. Cross-sectional studies can be used to describe endodontic disease prevalence (e.g. AP) as well as estimate its association with the quality of RCT/CR (i.e. concurrent exposure information) [24]. The study conducted by Petersson et al. established the general consensus that endodontic cross-sectional studies could provide reliable information on the long-term success rate of RCT at population levels [24][25].

Endodontic treatment and post treatment indexes (i.e. length and density of root filling and quality of CR) are the main factors that have a strong predictive effect on the outcome of RCT (presence/absence of AP) [22][26]. The prevalence of AP in endodontically treated teeth restored in the present study was 52%; concurring with the results of methodologically compatible cross-sectional studies in Brazil (50%) [22], Scotland (51%) [27], Canada (51%) [21], Denmark (52%) [15] and Turkey (53%) [14]. This prevalence was lower than those reported in Spain (65.8%) [12] and Germany (61%) [28], yet higher than those reported in Belgium (40%) [20], United States (39%) [11], Lithuania (39%) [29], France (33%) [30], Sweden (24%) [16], and Portugal (22%) [31]. This study indicated that AP is prevalent in the Iranian population and that RCT does not control the disease. Thus, the present study supported the well-documented conclusion that the realistic outcome of endodontically treated teeth in general population was significantly inferior to the potential outcome demonstrated in follow-up clinical studies.

In this study OPG radiographs were used for evaluating the quality of endodontically treated teeth. The radiographic measures of ‘length and density of root filling’ can be used as indicators to assess RCT’s capacity to prevent recontamination and it may substitute clinical measures that assess the quality of RCT. Unfortunately, the criteria for judging the quality of RCT have not been well defined. Acceptable RCT was defined as having ‘adequate length and density of root filling’. These subjective assessments have not been standardized or calibrated; however, the results of these subjective assessments showed that ‘acceptable RCT’ had significantly lower AP than those judged ‘unacceptable’ [26]. Furthermore, it has been contended that periapical diagnosis from OPGs may result in underestimation of the real prevalence of AP. However, research has indicated good association between OPGs and intra-oral radiographs, and even a slight overestimation [32][33][34]. It is therefore probable that the validity of recording AP based on OPGs is satisfactory.

Individuals with 10 or fewer remaining teeth were not included in this survey as they often had poor oral health and periodontal diseases and it was difficult to determine the influence of RCT on the incidence of radiographic AP [35][36].

Our sample consisted of more women than men (58.2% vs. 41.8%), which may form a recruitment bias, or indicate that female patients were more likely to seek dental care in the Iranian population. Other surveys also found a similar gender predilection [7][12].

However, gender had no significant effect on the presence of AP [26].

Younger patients (18-30 years) made up >60% of the sample, as shown in the age distribution table (Table 2). Other studies have also shown a similar distorted distribution [20][28][35][37][38], that could be because younger patients may more often seek dental treatment.

Our results showed that the number of teeth with AP were 527, representing ~1.9% of the total. The frequency of teeth with AP in other surveys varied from 0.6% [33] to 9.8% [39]. The reported frequency of AP seems very variable in different populations; this may be due to the variance in oral health as well as clinical skill of clinicians.

In 254 cases of endodontically treated mandibular molars, 61% presented with AP. The highest number of cases with AP was seen in mandibular first molars (112 cases). This tooth is the first to erupt in permanent dentition and therefore more prone to caries, trauma, operative intervention and pulp/periapical diseases.

Approximately 90% of periapical lesions were smaller than 3 mm. The size of the lesion may influence the decision to intervene, by both the patients and clinicians [40]. However, there is no difference in the outcome of endodontic treatment of teeth associated with small or large lesions. Large lesions require a longer time to heal, and therefore their assessment therefore requires longer follow up times [26].

The total percentage of endodontically treated teeth was ~3.5%, which is similar to other surveys (results ranged between 1.3-4.8%) [12][13][21][28][31][33][35]. However, some surveys found that the prevalence of endodontically treated teeth ranged from 8.6 to 26.0%, [7][10][29][36][38][41][42].

Our results demonstrated that the number of extracted teeth per person significantly increased with age; a common finding in all previous surveys. The mean number of remaining teeth in this survey was 26.75, again agreeing with cross-sectional surveys performed in other countries [7][13][21][29].

Some previously performed cross-sectional studies have shown relatively higher prevalence of missing teeth in their studied populations; this can could be due to the extraction of failed endodontically treated teeth with AP [10][20][31][38][42].

Recent epidemiological surveys have further investigated the significance of CR and suggested that the quality of the CR may affect the outcome of the RCT [22]. Our results demonstrated that apical periodontitis was present in approximately 40% of teeth which had a proper CR compared to 70% which did not have an adequate CR (P<0.001).

A direct correlation between quality of CRs and the presence/absence of AP was shown in this survey as well as many others [11][12][15][21][22][29][43]. Based on this finding, provision of the CR should be considered the final part of the RCT to prevent postoperative recontamination.

Apical periodontitis was 4.5 times more likely to be present in unacceptable RCTs, and 2.5 times more likely in inappropriate CR compared with appropriate ones. The most remarkable finding of this survey was related to the simultaneous effect of the quality of CR and RCT on AP. The odds of finding AP in unacceptable RCT/inappropriate CR cases was >11 times greater than acceptable RCT/appropriate CR. This finding has not been previously described in this way by epidemiological endodontic literatures.

## CONCLUSION

This cross-sectional survey of a selected Iranian population evaluated the quality of the RCT/CR in relation to the periapical status. Within the limitations of this study, the results demonstrated that a well-performed RCT and well-sealing CR are both essential for the overall success of endodontic treatment, concurring with almost all endodontic epidemiological surveys. Therefore we may conclude that a worldwide improvement in the quality of RCT/CR in general dental practice is required to promote oral/periapical health.
